# Supplementary material for: A systematic review of resilient performance in defence and security settings
Source: PLoS One. 2022 Oct 3;17(10):e0273015. doi: 10.1371/journal.pone.0273015 (PMC9529107; doi:10.1371/journal.pone.0273015)
Supplement: Attachment — Submitted filename: Response to Reviewers.docx [file pone.0273015.s003.docx]

Dear Dr Baker,

**RE: A systematic review of resilient performance in defence and security settings**

Many thanks for sending the reviewers comments on our manuscript. We are extremely grateful to you, and the reviewers, for the comments on our paper. By addressing these comments, we believe we have strengthened the final paper. Below we outline our responses to yours, and the reviewers’ comments in turn, and have amended the manuscript using the track changes function in Microsoft Word.

**Reviewer comments to address.**

| **Comment/Requirements** | **Comments/response** | **Location of changes** |
| --- | --- | --- |
| Editorial comments |  |  |
| Please ensure that your manuscript meets PLOS ONE's style requirements, including those for file naming. | We have checked the style templates and submission guidelines and have edited all documents to comply with the journals style requirements. | All documents submitted |
| Please note that in order to use the direct billing option the corresponding author must be affiliated with the chosen institute. Please either amend your manuscript to change the affiliation or corresponding author, or email us at plosone@plos.org with a request to remove this option. | All author and affiliation details have been provided and linked with the chosen institute. | This has been amended on the online submission portal and within the manuscript title page submission. |
| In your Data Availability statement, you have not specified where the minimal data set underlying the results described in your manuscript can be found. PLOS defines a study's minimal data set as the underlying data used to reach the conclusions drawn in the manuscript and any additional data required to replicate the reported study findings in their entirety. All PLOS journals require that the minimal data set be made fully available. | As this is a narrative systematic review submission, the data used has been extracted from the papers identified in the review process. We have provided the data extraction table as part of the manuscript submission. We do not have additional underlying results in the traditional sense and trust that this will suffice. If not please let us know what additional information is required. | No changes made. |
| PLOS requires an ORCID iD for the corresponding author in Editorial Manager on papers submitted after December 6th, 2016. Please ensure that you have an ORCID iD and that it is validated in Editorial Manager. | All author details have been updated, including providing ORCID ID’s on the editor manger page. | Details updated on the submission portal |
| **Reviewer 1.** |  |  |
| **1) Theoretical frameworks for measuring resilience.**  The authors were not able to make any clear conclusions due to the limited number of papers that included theory. As a whole, I don’t think this area adds much to the paper and may be worth removing. | This is a good point. We recognise that a limited number of papers were retrieved utlising a theoretical understanding of resilient performance however believe this point is worth noting. This is an important and emerging aspect of the literature and the finding that papers with theoretical underpinning is limited is an important finding within itself and therefore needs to be represented within this review. | We understand the reviewer’s comment, however no changes have been made in response. |
| **2) Measures associated with resilience.**  This section I feel the authors were most successful with. While the outcomes are quite varied in terms of the measures being used, the authors did a good job clustering them into groups to attempt to make a cohesive statement. | We thank the reviewer for the positive comments regarding this aspect of the review. | The comment requires no amendments to be made. |
| **3) Programs to improve resilience.** Again, there were limited (n=6) and varied studies in this area, which makes it difficult to make strong conclusions, although they do see some concordance in the area of mindfulness and other psychological skill training. | This is a good point and we recognise that there was limited research identified in this area. That said we have extracted common findings regarding use of mindfulness and psychological skills. This we believe is an important aspect to the current research in the area, and was a key research question for our aim. | We understand the reviewer’s comment however no changes have been made in response. |
| **4) The quality of the research related to the first three points.** The authors did a good job at rating the studies, as well as highlighting their strengths and weaknesses. | We again thank the reviewer for their positive response to this aspect of the paper. | No change required |
| At the end while a applaud the authors attempt to develop a framework, I feel that one individual’s attempt may likely get lost in the shuffle, or even make things more disjointed. This may be best done as a consensus statement by a major military psychological society (e.g. APA Society for Military Psychology). This may be worth removing. | The reviewer has highlighted an important point regarding work of this kind. However, the current work has been produced by an interdisciplinary group of researchers with extensive expertise in UK military research. Including some researchers employed in the UK Military of Defence (MOD). Furthermore, the research has been directly funded by the MOD. We recognise it is UK focused but believe that there is sufficient military input into the framework, certainly from a UK perspective. That said, we recognise the value of a consensus statement and hope that this review and model can feed into the development of such consensus statements. | We understand the reviewer’s comment however no changes have been made in response. |
| **Other specific comments**  - The process of citing a study by author is occasionally incorrectly done and confusing (e.g. Line 67). Please review and correct.  - There are a lot of leading phrases that are missing commas, which make the sentence difficult or confusing to read (e.g. Line 216, 231).  - Sentences on lines 208-211 don’t make sense.  - Line 355: grit and resilience are capitalized but not the other factors  - Line 357: missing parenthesis  - Line 404-407: These statements seem to be contradictory to each other. How can there be no difference, but also a small to medium effect size? Please clarify | We thank the reviewer for picking up on these errors in the paper. We have thoroughly proofread and amended all aspects of the manuscript. | Changes have been made throughout the manuscript highlighted in the marked-up copy of the resubmission. |
| **Reviewer 2.** |  |  |
| It is understood that there is no single biomarker that can be attributed to Physical, mental and cognitive performance. However, it seems some more highlights should be there regarding Biomarkers that can be connected to resilience of personnel in case of a security settings. This is very important because a biomarker can give a beforehand predictive information about a resilient performance that may be required in real scenario. | We thank the reviewer for recognising the importance of biomarkers on performance in this work. We understand that some more detail could be added regarding the connection between biomarkers and resilience. However, we also recognise we can only report the findings that have been picked up through the search criteria. The search criteria were extensively reviewed and piloted prior to use and developed by the research team in order to detect all papers relating to this aspect of the research. |  |
| Also, a schematic representation of the search strategy used (including databases and literature review) will be helpful for better understanding of the methodology adopted in this study. | A flow chart has been provided that outlines the review search process in line with the PRISMA checklist for systematic reviews | Please find the flow chart provided in support of this manuscript at Fig 1. |
| **Reviewer 3.** |  |  |
| 1. The review paper is completely theoretical and can be made more comprehensive for the readers by using some statistical techniques (charts, histogram etc.) by pooling the papers based on their subject and outcomes. | Because of the small number of papers retrieved from each category, a more in-depth statistical analysis was not deemed appropriate for the paper. | Details regarding the rationale for this decision of a descriptive approach has been provided within the manuscript. See lines 236-239. |
| 2. The review has not included research papers that have used various MCDM tools and techniques that have addressed the issues related to the resilient performance in defense and security.  3.The review has also not included research papers that have used various Human Reliability tools and techniques that have addressed the issues related to the resilient performance in defense and security. | We thank the reviewer for drawing our attention to other aspects of performance. The search terms developed for this systematic literature review reflect the aim of the review; to explore literature relating specifically to human aspects of resilient performance. However, our systematic search did not detect any papers using MCDM tools or Human Reliability tools in relation to resilient performance in defence and security settings. Given we have to stick to our systematic search criteria we were unable to include these papers in the review. | No changes have been made in response to this comment. |
| 4. The authors also need to explore more recent scientific work in this area. | The current systematic review covers papers from 2001 to February 2022, this is due to an update of the search as reported in the paper.  Therefore, we consider that the literature reported as part of the review is as up to date as feasibly possible at the date of paper submission. | No changes have been made in response to this comment. |
| 5. The suggested work as an extension may be based on certain latest MCDM and HR model that are not available in the literature. This can strengthen the contribution of the authors. | We again thank the reviewer for their consideration of other aspects of performance. However, as noted in response to comments 2 and 3, we suggest that the techniques suggested by reviewer 3 are outside of the scope of the current review. | No changes have been made in response to this comment. |
| 6. In its present form, this paper can be definitely deemed a "research review" but it needs to be more scientific in terms of available scientific models, the examples of which have been provided at points 2 and 3 , that addresses such issues and can be further extended to model a number of factors that have been exhaustively deliberated in this paper. | The aim of this narrative systematic review was to understand factors impacting resilient performance within defence and security personnel. Our pre-determined search criteria detected all existing literature relating to theories and models on this topic. The examples that you have provided did not meet our criteria for inclusion, - although are interesting - and consequently we have not included discussion of these in our systematic review. | No changes have been made in response to this comment. |

We would like to thank the reviewers again for their positive and helpful comments. We hope that these comments address the reviewers’ concerns.
